# Phage Therapy Related Microbial Succession Associated with Successful Clinical Outcome for a Recurrent Urinary Tract Infection

**DOI:** 10.3390/v13102049

**Published:** 2021-10-12

**Authors:** Austen Terwilliger, Justin Clark, Maile Karris, Haroldo Hernandez-Santos, Sabrina Green, Saima Aslam, Anthony Maresso

**Affiliations:** 1 TAILΦR Labs, Molecular Virology and Microbiology Department, Baylor College of Medicine, Houston, TX 77030, USA; terwilli@bcm.edu (A.T.); justin.clark@bcm.edu (J.C.); haroldo.hernandezsantos@bcm.edu (H.H.-S.); sabrina.green@bcm.edu (S.G.); 2 Center for Innovative Phage Applications and Therapeutics, Division of Infectious Diseases and Global Public Health, University of California, San Diego, CA 92093, USA; m1young@health.ucsd.edu

**Keywords:** phage therapy, microbial pathogenesis, UTI, humoral response, comparative genomics

## Abstract

We rationally designed a bacteriophage cocktail to treat a 56-year-old male liver transplant patient with complex, recurrent prostate and urinary tract infections caused by an extended-spectrum beta-lactamase (ESBL)-producing *Escherichia coli* (*E. coli*) (UCS1). We screened our library for phages that killed UCS1, with four promising candidates chosen for their virulence, mucolytic properties, and ability to reduce bacterial resistance. The patient received 2 weeks of intravenous phage cocktail with concomitant ertapenem for 6 weeks. Weekly serum and urine samples were collected to track the patient’s response. The patient tolerated the phage therapy without any adverse events with symptom resolution. The neutralization of the phage activity occurred with sera collected 1 to 4 weeks after the first phage treatment. This was consistent with immunoassays that detected the upregulation of immune stimulatory analytes. The patient developed asymptomatic recurrent bacteriuria 6 and 11 weeks following the end of phage therapy—a condition that did not require antibiotic treatment. The bacteriuria was caused by a sister strain of *E. coli* (UCS1.1) that remained susceptible to the original phage cocktail and possessed putative mutations in the proteins involved in adhesion and invasion compared to UCS1. This study highlights the utility of rationally designed phage cocktails with antibiotics at controlling *E. coli* infection and suggests that microbial succession, without complete eradication, may produce desirable clinical outcomes.

## 1. Introduction

Bacteriophages, the viral predators of bacteria, are the most numerous and prolific bacterial killers on earth. An estimated 10^31^ phages dispatch around 50% of 10^30^ bacterial cells in the world every day [1,2]. This enormous and constant genetic turnover provides near-infinite adaptive and evolutionary potential. When this landscape of possibility is compared to that of conventional antibiotics—with defined, rigid structures and limited chemical space—antibiotics just cannot keep pace with bacterial evolution that subverts, circumvents, and destroys them [3,4]. As the world enters the post-antibiotic era, phages—evolvable agents capable of great change—carry potential as a novel antibiotic approach and last-resort measure for persons suffering from multi-drug-resistant bacterial infections [5]. However, the application of these unique features encoded within phages have not been sufficiently explored in the scientific and medical setting with regard to efficacy and clinical outcome. Urinary tract infection (UTI) case studies employing phage therapy lack extensive mechanistic investigation or consideration for the biological context of the infection [6,7]. Scientific investigation of clinical cases remains essential to understanding the host/bacteria/phage relationship and developing effective phage therapies.

UTIs are common and frequently treated with antibiotics, with an estimated 11.3 million antibiotic prescriptions totaling $1.6 billion in annual costs in the US [8]. As 20% of persons with UTIs present to the emergency room for treatment, there are additional significant costs beyond the antibiotic prescription [9]. Further, a subset of persons experience complicated and recurrent UTIs, including pyelonephritis, sepsis, bacteremia, and prostatitis [10]. Recurrent UTIs are associated with biofilm (within urothelial mucosa, urinary stones, catheters); immunosuppression; and, in the case of prostatitis, the insufficient penetration of antibiotics [11,12,13]. *E. coli* is the most common pathogen associated with UTIs and increasingly presents as a multi-drug resistant organism (MDRO) [10].

Here, we report the use of a four-phage cocktail to treat complicated UTI. This unique cocktail included two phages that each bestowed a property potentially important for phage therapy. One phage binds human polysaccharides that are present at mucosal surfaces, a trait that could place the phage at the site of the bacterial infection [14]. Another phage, attained by directed evolution, targets the major bacterial pathway of phage resistance arising from the infection with another phage in the cocktail [15]. Thus, we developed a cocktail that is designed to both promote bacterial killing under physiologically relevant conditions (targeted phage localization) and reduce bacterial resistance by circumventing a common mechanism for *E. coli* phage evasion. Here, we report the patient’s outcome, immune response, and characteristics of the dominant residual bacterial strain that emerged following the cessation of therapy.

## 2. Materials and Methods

### 2.1. Human Subjects

As per the University of California San Diego’s (UCSD) Center for Innovative Phage Applications and Therapeutics (IPATH) protocol, we filed and received approval from the Food and Drug Administration for the emergency use of phage cocktail for the study participant. The UCSD Institutional Review Board and Institutional Biosafety Committee urgently approved use, and we obtained written informed consent from the participant.

### 2.2. Phage Discovery/Purification

All phages were first discovered as individual plaques on lawns of *E. coli* clinical isolates using the double agar overlay method [16]. We successively passaged each individual plaque by streaking onto fresh lawns twice, with single plaques taken each time. A single plaque from the final streak created a clonal plate lysate [16]. All phages were purified via CsCl gradient, as previously described [17], using the patient isolate UCS1 and titers determined by double agar overlay assay.

### 2.3. Phage Sequencing Analysis

Please refer to Supplemental Methods for phage genomic DNA preparation and sequencing. Raw reads were processed using FaQCs (version 2.09 with default settings, quality score cutoff of Q30, and reads less than 50 base pairs discarded) [18] and assembled using SPAdes (version 3.5.0) [19]. For each phage, a single circular contig produced the final assembly. Gene calling and annotation were done using the RASTtk pipeline (version 1.3.0, using default settings) [20] through PATRIC’s Genome Annotation service, [21] and tRNA predictions were done using ARAGORN (version 1.2.36.c) [22]. Coding sequences were searched for virulence and antibiotic resistance genes by using BLAST (version 2.8.1) [23] to compare assembled genomes against the Virulence Factor Database (VFDB; version 20200408) [24], the PATRIC virulence factor database [25], the Antibiotic Resistance Gene Database (ARDB; version 1.1) [26], and the Comprehensive Antibiotic Resistance Database (CARD; version 3.0.7) [27]. ShortBRED (version 0.9.4) [28] was used for targeted searches of coding sequences for genes in VFDB, CARD, and the Resfam antibiotic resistance gene database [29]. Phage genus was predicted from sequenced relatives identified by using BWA-Mem (version 0.7.9) [30] by aligning contigs to NCBI’s RefSeq database and by CDS homology using PHAge Search Tool Enhanced Release (PHASTER) [31]. Phage lifestyles were predicted using PHACTs [32]. Integrases and attachment sites were queried using PHASTER and by parsing annotated genomes for “integrase”. The percentage of total reads mapped to the host was determined by aligning reads to NCBI’s RefSeq database using BWA-Mem and determining the number of reads that mapped to an *E. coli* genome over the total number of reads.

### 2.4. Bacterial Genome Assembly and Analysis

Please refer to Supplemental Methods for bacterial genomic DNA preparation and sequencing. Raw reads were trimmed using BBDuk (version 38.84 with default settings, quality score cutoff of Q30, and reads less than 50 base pairs discarded) and initially assembled de novo using SPAdes (version 3.5.0). Contigs were aligned using Mauve Contig Mover (version 2.4.0) and classified using tools available from the Center for Genomic Epidemiology: Multi-Locus Sequence Typing 2.0 (software version 2.0.4 and database version 2.0.0), SeroTypeFinder (software version 2.0.1 and database version 1.0.0), and FimTyper (version 1.0) [33,34,35,36]. A custom database of virulence factors and the Comprehensive Antibiotic Resistance Database (CARD; version 3.0.7) were used as databases for megaBLAST (version 2.8.1) runs using the contigs as a query to determine the presence of ExPEC-associated virulence and antibiotic-resistance factors [37]. To limit the number of spurious and repeat hits, only the top High-Scoring Segment Pairs (HSP) were kept by setting the “culling_limit” and “max_hsps” to “1”. The resulting identity heatmaps were created using Graphpad Prism version 9.2.0 for Windows, GraphPad Software, San Diego, California USA, www.graphpad.com accessed 12 September 2021. The heatmaps are presented in Figure S1. Based on results from this, EC958 was chosen as a reference to map raw reads using Geneious assembler in Geneious Prime 2019.2.3 (www.geneious.com accessed 12 September 2021) using medium/fast sensitivity and settings to detect polymorphisms of any size and type. The resulting assemblies were annotated using RASTtk (version 1.3.0) using default settings [20,38,39]. The annotated UCS1 and UCS1.1 genomes (Accession CP084679 and CP084678, respectively) were then aligned in Mauve (version 2.4.0) and disagreements extracted using Geneious SNP and Variant Finder using default settings with a minimum variant frequency of 0.50 (Figure S2).

Phylogenetic analysis was performed using The Automated Multi-Locus Species Tree (autoMLST) software [38]. AutoMLST uses Multi-Locus Species Analysis (MLSA) on up to 100 housekeeping genes present in all strains of interest. We used default settings with IQ-TREE Ultrafast Bootstrap analysis to create a concatenated tree based on genes selected automatically. The resulting trees were imported into Geneious for visualization and editing. MLST gene list, identity matrixes, and MASH/Average Nucleotide Identity (ANI) can be found in Supplementary Material. Results were validated by determining the core SNP differences between the strains using Snippy [39] (see Supplementary Material).

### 2.5. Cocktail Formulation

The calculated concentration of each phage in the cocktail was as follows: HP3–7.5 × 10^9^ plaque forming units (PFU)/mL (Accession KY608967); HP3.1–1.35 × 10^10^ PFU/mL (Accession OK275722); ES17–1.37 × 10^10^ PFU/mL (Accession MN508615); ES19–3.35 × 10^9^ PFU/mL (Accession MN508616). Endotrap HD Column (Lionex GmbH) removed endotoxin following manufacturer’s instructions for column processing.

### 2.6. Killing Assays 

We grew UCS1 cells to log phase, then seeded 10^7^ CFU’s into 100 µL lysogeny broth (LB) media of each well in a 96-well plate. Using the reported titer of 3.0 × 10^10^ PFU/mL, the phage cocktail was added to the wells at the indicated multiplicity of infection (MOI) and incubated/shaken at 37 °C for 4 h. Serial dilutions of the cultures and the starting inoculum (control) were then plated using 10 µL slants on LB agar plates. The plates were incubated at 37 °C overnight and the resulting colonies counted. 

### 2.7. Luminex Data Analysis and Heatmap Creation

Patient sera samples before, during, and after phage administration were subjected to antibody-based quantification with a Milliplex human 48-plex cytokine panel (Cat# HCYTA-60K-PX48) according to the manufacturer’s protocol. Data were generated with Luminex xPONENT for Magpix, version 4.2 build 1324 on a Magpix instrument and analyzed with Milliplex Analyst, version 5.1.0.0 standard, build 27 October 2012. Results were considered statistically significant with ≥35 counts per analyte per well. The median fluorescent intensity for standard curves was analyzed using a 5-parameter log curve-fitting method to calculate sera concentrations. Concentrations (pg/mL) of analytes were determined by averaging duplicate samples and standardized to concentrations of samples taken before phage treatment was administered (pretreatment levels). Values were base 2 log transformed (log_2_) and processed with Heatmapper to create heat maps and hierarchal clustering [38]. Hierarchal clustering was done with average linkage clustering method and Euclidean distance measurements.

### 2.8. Synography

Antibiotic-phage synograms were performed as previously described by our lab [40]. Briefly, each well of a 96-well plate was inoculated with 5 × 10^7^ CFU of UCS1 and subjected to increasing concentrations of drug, phage cocktail, or both in LB and incubated/shaken at 37 °C for 24 h.

### 2.9. Serum Neutralization

A UCS1 cocktail mimic was prepared by mixing phages HP3, HP3.1, ES17, and ES19 from the same stocks as the original cocktail. Serum samples were serially diluted 1:10 and 1:100 in phage buffer. 100 µL each of serum and diluted samples were mixed with 1 µL of the cocktail mimic in a 96-well flat bottom plate and incubated/shaken at 37 °C for 30 min. Serial dilutions of each well were spotted onto a UCS1 lawn in a double agar overlay assay and resulting PFUs counted to determine titer.

## 3. Results

### 3.1. Participant Characteristics and Phage Therapy Design Modality

The phage cocktail recipient was a 56-year-old man with well-controlled human immunodeficiency virus (HIV) infection and hepatitis B virus infection who was status post-liver transplant and on tacrolimus since 2014. He had symptomatic recurrent prostatitis and UTIs in the setting of kidney stones from April 2017 to January 2020 due to ESBL-producing *E. coli*, for which he completed multiple courses of intravenous (IV) ertapenem for varying 6–8 week durations, with several courses including the concurrent use of azithromycin and trimethoprim/sulfamethoxazole based on antibiotic synergy testing and enhanced prostate tissue penetration. He also completed multiple courses of oral fosfomycin and N-acetylcysteine for biofilm dissolution. He additionally underwent the surgical removal of his kidney stones and lithotripsy, although small punctate irremovable stones remained. He did not tolerate gentamicin bladder washes and, in general, symptomatic UTI recurred within 2–3 weeks of antibiotic discontinuation. Phage therapy provided a last resort option in an effort to resolve the infection.

TAILΦR (Tailored Antibacterials and Innovative Laboratories for Phage (Φ) Research) received the UCS1 ESBL-producing *E. coli* clinical isolate on an agar slant from IPATH. It exhibited the typical green sheen on Eosin Methylene Blue (EMB) agar, indicative of lactose fermentation (Figure S3). Our *E. coli* phage library was screened via spot assay and identified that 20 of the 22 phages tested formed clearings. The details of the four selected phages are noted in Table 1.

Phages HP3, ES17, and ES19 were previously characterized by Gibson et al. [41]. Their genomes are devoid of undesirable elements (toxins, antibiotic resistance, and integrase genes). ES17 was further investigated by Green et al. [14]. Through selective screening, they discovered that ES17 possesses mucolytic traits and binds to heparan sulfate proteoglycans (HSPGs). These features promote infection in mucoid environments, such as the gut. Phage ES17 is particularly unique in that it binds to bacterial and human carbohydrates, which may positionally target the phage to unique mucosal environments where the bacteria localize [42].

### 3.2. Safety and Efficacy of Phage Cocktail

**Figure 1:**
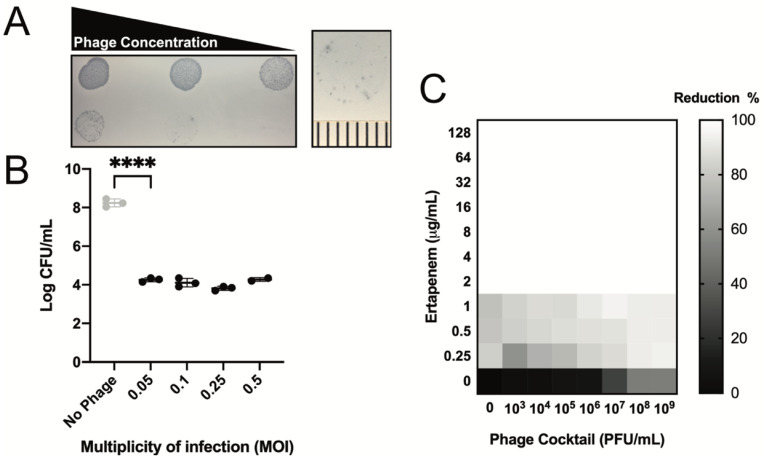
Characterization of UCS1 Cocktail. (**A**) Serial dilution of UCS1 Cocktail onto lawn of UCS1 *E. coli*. Each tick mark represents 1 mm. (**B**) Killing Assay. 10^6^ CFUs of UCS1 *E. coli* were incubated without phage or indicated MOI of UCS1 cocktail for 4 h in 100 µL LB, and resulting CFUs counted. Error bars represent mean +/− SD n = 3 technical replicates **** *p* < 0.001. (**C**) Synogram performed as described in Gu Liu et al. 2020 [39]. Each well of a 96-well plate was inoculated with 5 × 10^8^ CFU of UCS1 and subjected to increasing concentrations of drug, phage cocktail, or both in LB and incubated/shaken at 37 °C for 24 h. Optical Density 600 nM measurements were taken every 30 min. Values are reported as % reduction in growth at 24 h compared to untreated controls.

Where possible, we employed consensus best practices to guide the cocktail formulation and characterization [43]. In vitro safety was assayed via three measures: genomic analysis, endotoxin quantification, and sterility, with the results presented in Table 1. The concentration of endotoxin units delivered per 10^9^ PFU was determined to be 22.83 EU/dose, well within the 5 EU/kg/hr limit appropriate for the patient (weight 68.6 Kg) [44], and USP 71/AFB testing demonstrated the sterility of the final product. The final cocktail of four phages was highly lytic in both the plate and broth assessments of killing activity (Figure 1A,B). All the MOIs appeared to reduce the bacterial CFU burden by 4 logs when compared to the uninfected control. We performed synography to assess the ability of the phage cocktail to synergize with the ertapenem. Slight antagonism was observed at the lowest concentrations of ertapenem (0.25 µg/mL) and phage (10^3–5^ PFU/mL) tested, with an additive effect observed at higher phage concentrations (10^7–9^ PFU/mL) (Figure 1C). Taken together, these data suggested that the UCS1 cocktail was virulent and suitable for the combinatorial treatment with ertapenem.

### 3.3. Phage Treatment: Clinical Safety and Efficacy

**Figure 2:**
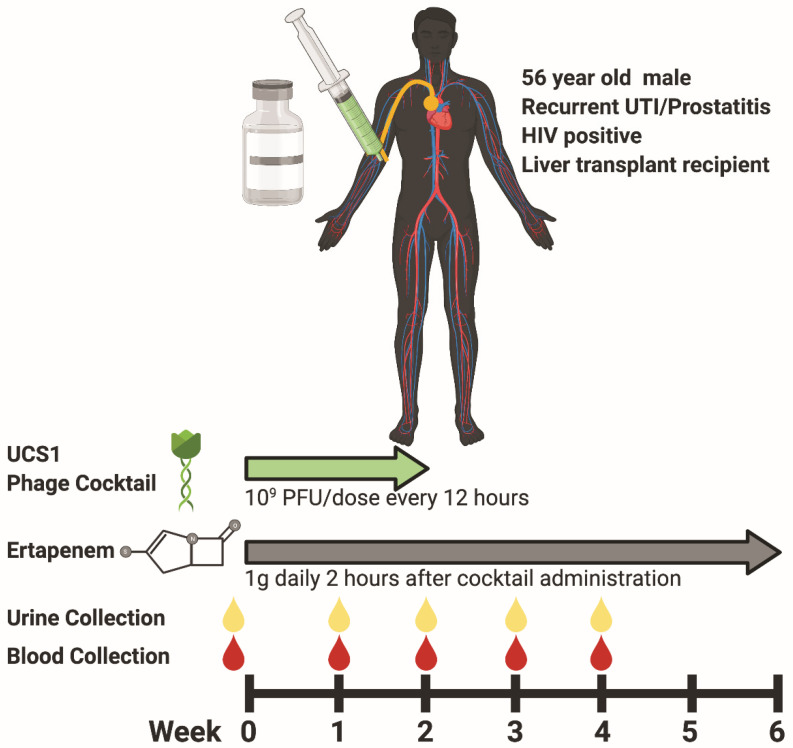
Treatment and clinical sampling course for patient with ESBL-producing *E. coli* UTI. (**Top**) Patient overview, administration route (PICC line, orange), and treatments (UCS1 cocktail, green; ertapenem, gray). (**Bottom**) Time course for dosing regimen of UCS1 cocktail (green), ertapenem (gray), and clinical sampling of urine (yellow drops) and blood (red drops). Created with BioRender.com accessed 23 September 2021.

The patient received two weeks of intravenous (IV) phage therapy from 2/4/20 to 2/18/20 as well as six weeks of IV ertapenem, ending on 3/16/20. Each dose consisted of 1.0 × 10^9^ PFU/mL of the phage cocktail every twelve hours. The once-daily ertapenem was given two hours after the morning phage dose (Figure 2). The first dose of phage was administered in the inpatient setting with frequent monitoring of the vital signs. After training and with self-administration instructions, the remaining doses were administered by the patient via a peripherally inserted central catheter at home. The patient kept a daily symptom diary, had weekly clinic visits with blood draws for the assessment of liver and kidney function, complete blood counts, and inflammatory markers. The patient tolerated the phage infusions well without any clinical adverse events and no concern for organ rejection. The liver and kidney function and complete blood counts remained stable. The C-reactive protein remained normal, while the sedimentation rate declined from 83 to 48 mm/hr. We collected baseline and weekly serum trough and urine samples while on phage therapy. No bacteria were detected in the urine after the first dose of the phage and ertapenem. At the end of therapy (EOT) on 3/16/20, the patient did not have any symptomatic recurrence of UTI in the 12 weeks of follow-up. However, the surveillance urine cultures were positive for asymptomatic bacteriuria at weeks 8 and 13 (2 and 7 weeks following EOT) for which antibiotic treatment was not needed.

### 3.4. Longitudinal Assessment of Humoral Response

**Figure 3:**
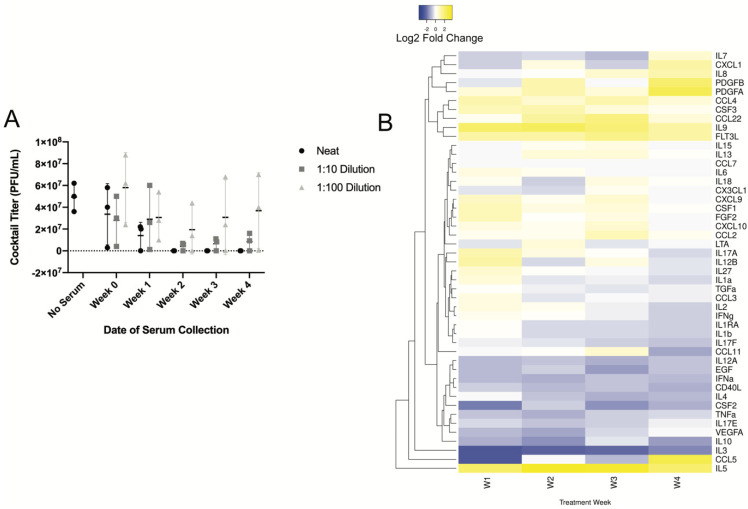
Patient humoral response to UCS1 Cocktail. (**A**) Equal volumes of the UCS1 cocktail mimic were mixed with phage buffer alone (No Serum), weekly patient serum samples (Neat), or dilutions in phage buffer and incubated/shaken at 37 C for 30 min. Serial dilutions from each condition were spotted onto lawns of UCS1 *E. coli* to determine titer. Results are reported as average +/− SD. n = 3 technical replicates. (**B**) Luminex assays performed on weekly sera samples. Concentrations (pg/mL) of humoral markers were measured in duplicate, averaged, and standardized to pretreatment levels before being converted to Log2 values. Heatmap and hierarchal clustering were done using HeatMapper with average linkage clustering method and Euclidean distance measurements.

We found that the patient’s sera from weeks 1–4 led to the complete in vitro neutralization of the phage activity when assessed using the patient’s *E. coli* isolate (Figure 3). This effect was rescued by serum dilution, indicating the inhibitory component was a part of the serum.

When compared to serum levels prior to treatment, humoral analytes fell into four categories based on hierarchical clustering: increased, decreased, decreased following increase, and fluctuated (Figure 3B). Most prominently, interleukin-5 (IL-5) increased up to four-fold across weeks 1–4 (2 weeks during and 2 weeks after phage therapy). An increasing pattern was observed for platelet-derived growth factors A and B (PDGFA, PDGFB) and chemokine C-C motif ligand 4 (CCL4) for all 4 weeks, while IL9, IL-7, and IL-8 only increased after the completion of the phage administration (weeks 3–4). Several analytes increased in the first week of phage therapy, then decreased the following 3 weeks, including IL-12B, IL-27, IL-1a, transcription growth factor A (TGFA), CCL3, IL-2, and interferon gamma (IFNy). The fluctuating analytes—ones that changed weekly without a clear discernable pattern—included IL-15, IL-13, IL-6, IL-18, C-X3-C motif chemokine ligand 1 (CX3CL1), C-X-C motif ligand 9 (CXCL9), CXCL10, C-C motif ligand 7 (CCL7), and CCL2. Finally, the analytes that decreased across all 4 weeks compared to the pre-treatment (week 0) sera included IL-10, IL-4, IL-3, IL-12A, epidermal growth factor (EGF), IFNa, tumor necrosis factor alpha (TNFa), and vascular endothelial growth factor A (VEGFA).

### 3.5. Assessment of ESBL-Producing E. coli Strain Following Phage Therapy Treatment

**Figure 4:**
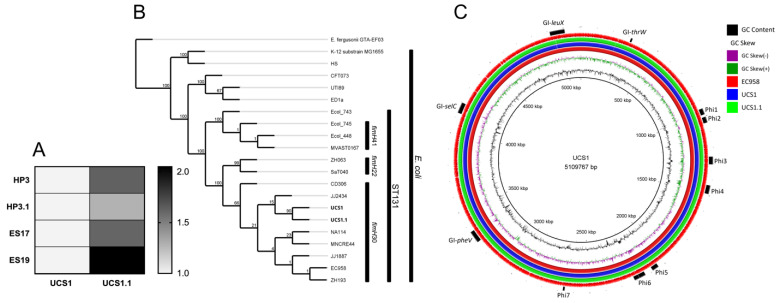
UCS1 and UCS1.1 are nearly identical ST131 strains. (**A**) Purified phages HP3, HP3.1, ES17, and ES19 were tittered onto lawns of UCS1 and UCS1.1. Efficiency of Plating (EOP) was calculated using UCS1 as the reference strain (EOP = 1). n = 3 technical replicates. (**B**) Phylogenetic tree was generated using autoMLST and visualized using Geneious Prime. UCS1 and UCS1.1 are shown in the context of other ST131 strains from the three major subtypes (*fimH*22, *fimH*30, and *fimH*41) and strains from other relevant UPEC sequence types: CFT073 (ST73) and UTI89 (ST95). Nodes are annotated with Ultrafast Bootstrap values. (**C**) Circular genome visualization created using BLAST Ring Image Generator (BRIG). EC958 acted as reference genome. Rings from inner to outer are as follows: (1) GC Content, (2) GC skew, (3) *E. coli* EC958 (Red), (4) *E. coli* UCS1 (Blue), (5) *E. coli* UCS1.1, and (6) CDS features (from EC958).

The surveillance urine culture at week 8 (six weeks after phage therapy cessation, two weeks after EOT) grew ESBL-producing *E. coli* (labeled here UCS1.1). The repeat urine culture at week 13 also grew ESBL-producing *E. coli*. Furthermore, UCS1.1 was just as susceptible to the original phage cocktail as UCS1 (EOP’s~1, Figure 4A), suggesting the UCS1.1 may be equivalent to UCS1.

To test this hypothesis, we sequenced both UCS1 and UCS1.1 and performed comparative genomics. The analysis revealed both are O25b:H4:K100 strains belonging to the *fimH*30 subgroup of the ST131 sequence type, one of the most prevalent ExPEC-causing sequence types in the world and currently responsible for the ST131 pandemic [45,46]. UCS1 and UCS1.1 contained many ExPEC-associated virulence factors, including *papA*, *afa* fimbriae, yersiniabactin (*fyuA*), aerobactin (*iutA*), *iss,* and *iha* (Figure 4B). Compared to other ST131s, the de novo assembled genomes had a serotype, *fim-*type, and virulence profile most similar to EC958, a UPEC strain isolated in 2005 [47]. This was the basis for using EC958 as a reference to assemble the genomes for direct comparison. The final assemblies showed that UCS1 and UCS1.1 contained all the Genomic Islands (GI-*thrW*, GI-*pheV*, GI-*selC*, GI-*leuX*) and prophages (Phi1-7) described in EC958 (Figure 4C) [47].

There were dozens of SNPs, insertions, and deletions that distinguished the two strains, including putative mutations in the attachment, motility, and secretion system elements. These changes are listed in Table 2. The effect of these changes on the virulence of UCS1 and UCS1.1 is unclear.

## 4. Discussion

UTIs are a common cause of recurrent infection and are associated with the development of multi-drug resistance. We describe the successful resolution of a symptomatic, recurrent, and complicated ESBL-producing *E. coli* UTI in an immunosuppressed patient treated with combinatorial phage cocktail and ertapenem. Our study affirms the clinical safety of phage therapy when administered intravenously at a dose of 10^9^ PFUs/mL, as well as the safety of self-administration, as noted previously [48,49,50]. An additional key finding was the onset of serum neutralization of the phages 1 week after treatment initiation, which has implications for phage therapy duration as well as the consideration of other routes of phage instillation, including intra-vesical for UTI. The baseline serum did not inhibit the phage infectivity; however, the sera collected at week 1 greatly reduced plaque formation and completely abolished phage infectivity from week 2 onward. Sera inhibition at week 1 suggests the action of an innate immune response, while the inhibition at later weeks is consistent with an adaptive response. This result agrees with previous studies investigating the presence of neutralizing antibodies in response to phage therapy [51].

We also utilized antibody-based detection assays to quantify humoral analytes before, during, and after phage administration. Most notably, IL5 exhibited the highest increase during and after treatment. IL5 is produced by Th2 helper cells and stimulates B-cell development and IgA secretion [52]. Likewise, IL7 and growth factors PDGFA, PDGFA, and IL8 levels appeared to increase the B-cell development, and the IgA secretion would agree with the increased mucosal immune activity in the urinary tract and observed serum neutralization of the phage cocktail [53]. However, other B-cell-associated factors, such as IL2, IL4, IL12, and IFNγ, decreased, along with pro-inflammatory markers, such as IL1α, IL1β, TNFα, and IFNα. This may be attributable to the concurrent provision of tacrolimus, a calcineurin phosphatase inhibitor used for immunosuppression for transplant recipients. Overall, the results of the serum analysis are consistent with an induced adaptive immune response to the UCS1 phage cocktail.

The patient had asymptomatic bacteriuria following EOT, unlike previous symptomatic recurrences requiring antibiotic courses the prior year, suggesting the presence of a non-pathogenic bacterial isolate. This bacteriuria was caused by a nearly identical strain of ESBL-producing *E. coli* (UCS1.1). The putative mutations noted in UCS1.1 may provide possible mechanisms for the altered pathogenicity. The prophage-associated invasin-like protein beta-barrel domain shares homology with the beta-barrel domain of FdeC (19% amino acid homology overall), an adhesin that mediates biofilm formation and is necessary for kidney colonization in the mouse urinary tract [54]. The immunization with FdeC also conferred protection against a murine model of UTI, making it a strong UPEC vaccine candidate [55]. The Vgr proteins are components of the baseplate and tip structures of the type VI secretion systems (TSS). TSS complexes mediate biofilm formation and interspecies competition for colonizing microbiome niches. Similarly, colicin immunity protein prevents colicin from destroying the cell’s own membrane [56]. Ultimately, UCS1.1 may have lost or altered these factors that confer adherence, invasion, and biofilm formation abilities to UCS1.

The genomic analysis indicated that UCS1.1 originated alongside and was derived from UCS1 rather than an independent infection event. Thus, it is intriguing that UCS1.1 was the predominant isolate after the phage therapy. The near identity of the clinical isolates and continued recurrence may indicate the presence of quiescent intracellular bacterial communities [57,58], a condition that would impede complete microbial clearance. UCS1.1 could be a rare variant or simply the result of genetic drift. UCS1 was likely cleared by the phage/antibiotic combination treatment, leaving the microbial niche to be filled by UCS1.1. A rare variant or genetic drift would also explain why UCS1.1 is just as susceptible to the phages in the UCS1 cocktail. Rather than develop resistance to the phage cocktail, microbial succession and expansion occurred once the predominant ESBL-producing *E. coli* was cleared. It is also possible that the mutations in UCS1.1 do not appreciably affect virulence when compared to UCS1. In that case, the patient’s improved clinical presentation could be due to biofilm reduction and/or alteration of the urobiome. This hypothesis is congruent with phage disruption of biofilms and dysbiosis-driven UTI [59,60,61]. Such an observation is consistent with our phage selection based on anti-biofilm and anti-resistome activity. Future efforts will focus on reverse genetics and murine models of UTI to test potential mechanism(s) of virulence attenuation.

Due to their prevalence, penchant for multi-drug resistance, and easy access for drug delivery, UTIs are desirable targets for phage therapy trials. The previous phage therapy for UTI trials evaluated the defined cocktails. However, a recently initiated study [62] will evaluate pre-approved phages that are variably matched to each patient’s clinical bacterial isolate, similar to the “sur-mesure” personalized approach [63]. Our case report supports the use of a rationally designed phage cocktail with antibiotics to treat recurrent UTI and demonstrates that, in addition to complete bacterial eradication, favorable microbial succession should be considered a desirable clinical outcome.

## Figures and Tables

**Table 1:** Composition, Genomic Analysis, and Properties of UCS1 *Escherichia coli* Phage Cocktail.

Bacteriophages	HP3	HP3.1	ES17	ES19
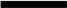 100 nm	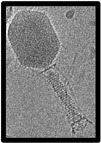	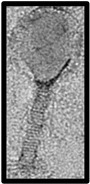	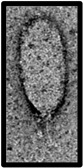	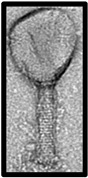
**Characteristics**				
**Morphology**	Myovirus	Myovirus	Podovirus	Myovirus
**Source**	Goose/Duck Feces	Directed Evolution	Human Sewage	Human Sewage
**Genome Size (bp)**	168,188	168,195	75,134	167,088
**Coding Sequences (CDS)**	264	266	124	263
**Hypothetical Proteins**	26	26	79	24
**Proteins with Functional Assignment**	238	240	45	239
**Closest Sequenced Relative**	Escherichia virus T40	Escherichia virus T40	Escherichia phage Eco32	Escherichia phage HY01
**Antibiotic Resistance CDS**	NO	NO	NO	NO
**Bacterial Virulence CDS**	NO	NO	NO	NO
**Attachment Sites**	NO	NO	2	NO
**Integrases**	NO	NO	NO	NO
**Lifestyle Prediction**	Lytic	Lytic	Likely Lytic	Lytic
**Titer in Cocktail (PFU/mL)**	7.5 × 10^9^	1.35 × 10^10^	1.37 × 10^10^	3.35 × 10^9^
**Cocktail Properties**				
**Date of Manufacture**	10/23/19			
**Titer (PFU/mL)**	3 × 10^10^			
**Endotoxin Content (EU/mL)**	685			
**Endotoxin Units per Dose (EU)**	22.83			
**Storage**	4 °C in 2.5 mL Glass Vial			
**USP 71 Sterility Testing**	No Growth Detected			
**Acid Fast Bacteria Testing**	No Growth Detected			

Phages HP3, ES17, ES19 were imaged and their genomes analyzed in Gibson et al. [41]. HP3.1 was imaged according to those same methods. All genomes were further analyzed according to methods above. Certified testing of endotoxin, sterility, and mycobacteria were performed according to USP guidelines, and certificates of analysis are presented in Figure S4.

**Table 2:** Notable Mutations in UCS1.1 compared to UCS1.

Coding Sequence	Locus Tag	Length (bp)	Type	Amino Acid Change	Protein Effect
Probable lipoprotein YPO0703	EC958_0641	3	Deletion		Extension
Prophage-associated invasin-like protein	EC958_1349	0	Insertion		Truncation
Prophage-associated invasin-like protein	EC958_1349	2	Deletion		Frame Shift
Prophage-associated invasin-like protein	EC958_1349	4	Substitution		Extension
Large exoproteins involved in heme utilization or adhesion, upaH	EC958_1689	9	Deletion	NTGT → N	Deletion
Glutamate decarboxylase, gadB	EC958_1756	5	Deletion		Frame Shift
Putative capsular polysaccaride transport protein, YegH	EC958_2403	1	SNP (transition)	I → M	Substitution
Glutamate decarboxylase, gadA	EC958_3921	1	Insertion		Extension
Glutamate decarboxylase, gadA	EC958_3921	0	Insertion		Frame Shift
SisB (ShiA homolog)	EC958_4074	1	SNP (transversion)	Q → L	Substitution
Uncharacterized protein YeeP	EC958_4120	2	Deletion		Frame Shift
Antigen 43	EC958_4121	2	Insertion	N → NAATNVT	Insertion
Anti-adapter protein IraM	EC958_4960	7	Deletion		Extension
Antigen 43	EC958_5138	0	Insertion		Frame Shift
Microcin-E7 immunity protein	EC958_5152	2	Deletion		Frame Shift
Small inner membrane protein, YmgF family	NA	3	Substitution	V → R	Substitution

Contig assembly and genomic analysis were performed as described in Methods and Figure 4. Mutations in transposable elements were removed, along with genes of unknown function, or for which no protein effect was predicted. Genes that code for known and putative virulence factors, as well as genes involved in nutrient uptake, are presented here. Gene designations are listed for strain EC958, the closest ancestral strain. Deletions resulting in large protein truncations are omitted from “Amino Acid Change” for clarity.

## Supplementary Materials

The following are available online at www.mdpi.com/article/10.3390/v13102049/s1, Supplemental methods, Figure S1. Comparative pathogenomics and resistomics of UCS1 and UCS1.1 with other *E. coli* species, Figure S2: Bioinformatic pipeline for genomic comparison between UCS1 and UCS1.1, Figure S3: Colony Morphology of UCS1 *E. coli*, Figure S4: Characterization of UCS1 cocktail by certified laboratories, Full mutation list.xlsx, auto MLST Snippy Data.xlsx, and Concatenated autoMLST.fasta.
