# Supplementary material for: Phage Therapy Related Microbial Succession Associated with Successful Clinical Outcome for a Recurrent Urinary Tract Infection
Source: Viruses. 2021 Oct 12;13(10):2049. doi: 10.3390/v13102049 (PMC8541385; doi:10.3390/v13102049)
Supplement: Supplementary file 1 [file viruses-13-02049-s001.zip › viruses-1374943-supplementary.pdf]

## Supplemental Methods

**Endotoxin Quantification** The UCS1 *E. coli* phage cocktail was serially diluted 3 logs ($10^{-3}$) in endotoxin-free water so that the endotoxin concentration fell within the range of the Endosafe Cartridge (PTS201, 0.1-1 EU/mL), then submitted to the Cell and Gene Therapy Center at Baylor College of Medicine for analysis. A Limulus Amoebocyte Assay (LAL) was performed on the diluted cocktail using a PTS201 Endosafe cartridge. The analysis certificate is in **Figure S4**.

**Sterility Testing** 100µL of UCS1 phage cocktail from each aliquot was sent to NOVA biologicals (Conroe, TX) for USP 71 sterility testing, with 50µL each inoculated into Fluid Thioglycollate medium and Tryptic Soy Broth. 100µL of the cocktail was also sent to NOVA for Acid Fast Bacteria (mycobacteria) testing. The analysis certificates are in **Figure S4**.

**Genomic DNA preparation and sequencing**

Phage genomic DNA was extracted and sequenced as described in Green et al 2017 [17]. Paired-end libraries were constructed from purified DNA. Amplicons were visualized via gel electrophoresis and purified to remove primer-dimers and non-specific amplicons (ChargeSwitch PCR Clean-up Kit, Invitrogen). Quantification utilized the Qubit® Fluorometer resulting in pooled samples at DNA mass of 100 ng per sample. The amplicon pool was sequenced on the Illumina MiSeq platform using reagent kit v2 (2 × 250 bp) paired-end protocol yielding paired-end reads that overlap almost completely.

Bacterial genomic DNA was purified using Omega E.Z.N.A. Bacterial DNA Kit (Cat#D3350) according to manufacturer's protocol, and sequencing performed by Novogene Corporation Inc.

## Supplementary Figures

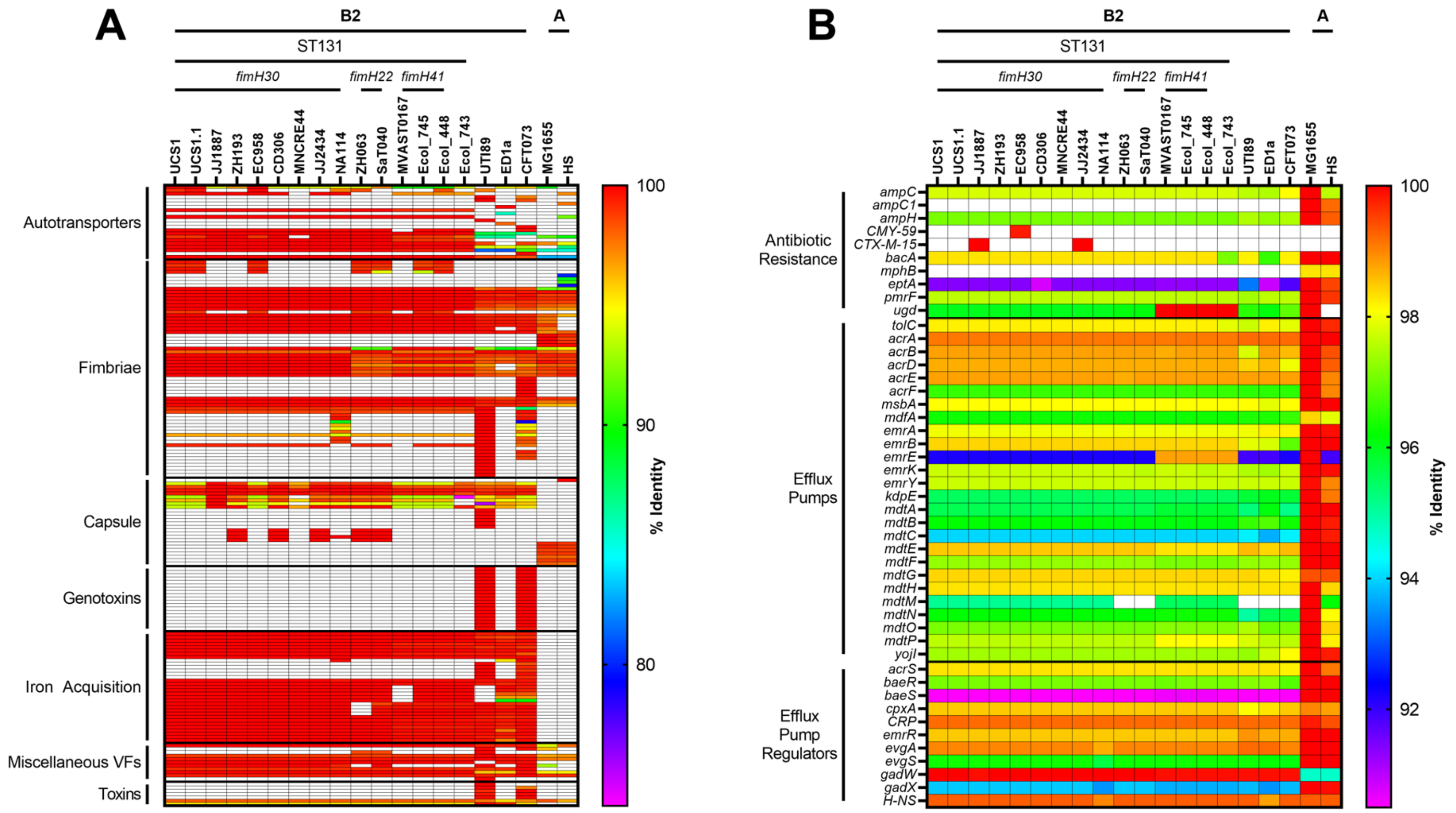


**Figure S1.** Comparative pathogenomics and resistomics of UCS1 and UCS1.1 with other *E. coli* species**.**

Several ST131 strains, including archetypal members of well-known ExPEC-associated ST95 (UTI89) and ST73 (CFT073) sequence types, and 3 non-pathogenic strains (ED1a, HS, and K-12 MG1655) were used as queries against either a custom *E. coli* virulance factor (VF) database (**A**) [37] or against the Comprehensive Antibiotic Resistance Database (CARD) (**B**). Heatmaps show the percent identity relative to the reference gene in the database. To generate the heatmaps with Graphpad Prism 9.2.0, megaBLAST aligned VFs or antibiotic resistance genes against each strain. The percent identity of High-scoring Segment Pairs (HSPs) that covered >90% of the reference gene was extracted and mapped.

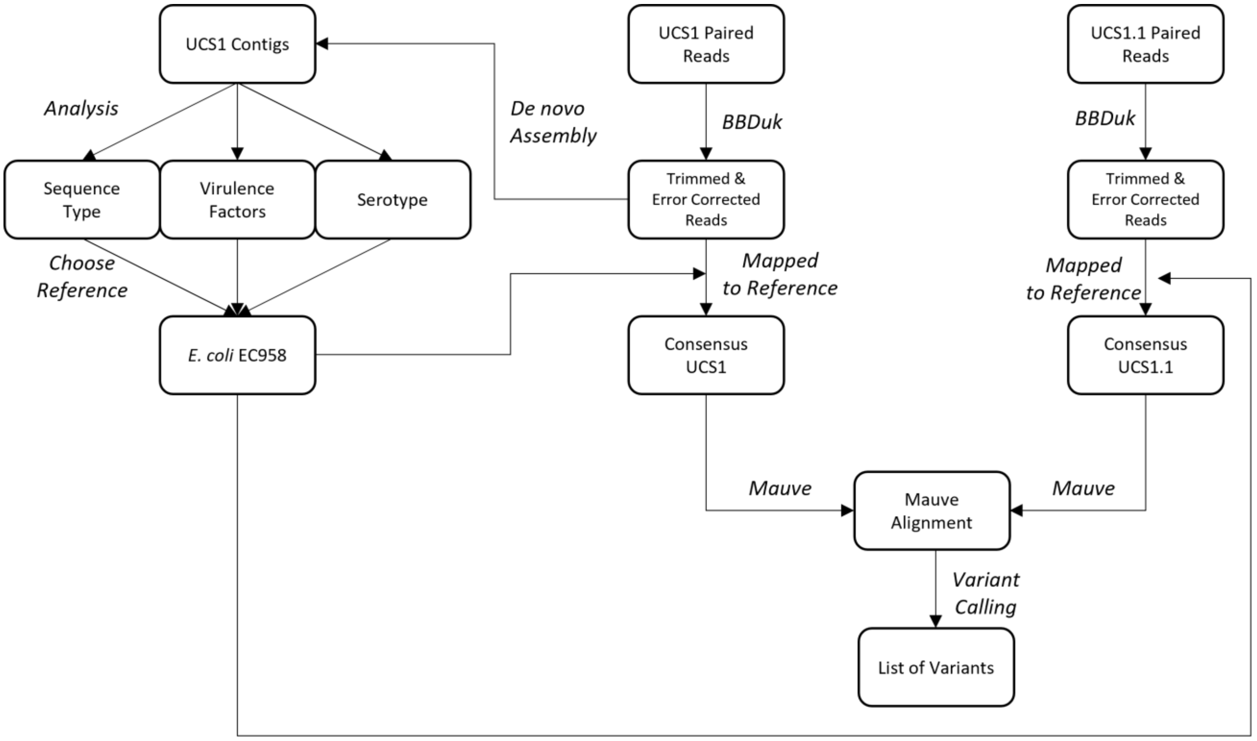


**Figure S2.** Bioinformatic pipeline for genomic comparison between UCS1 and UCS1.1

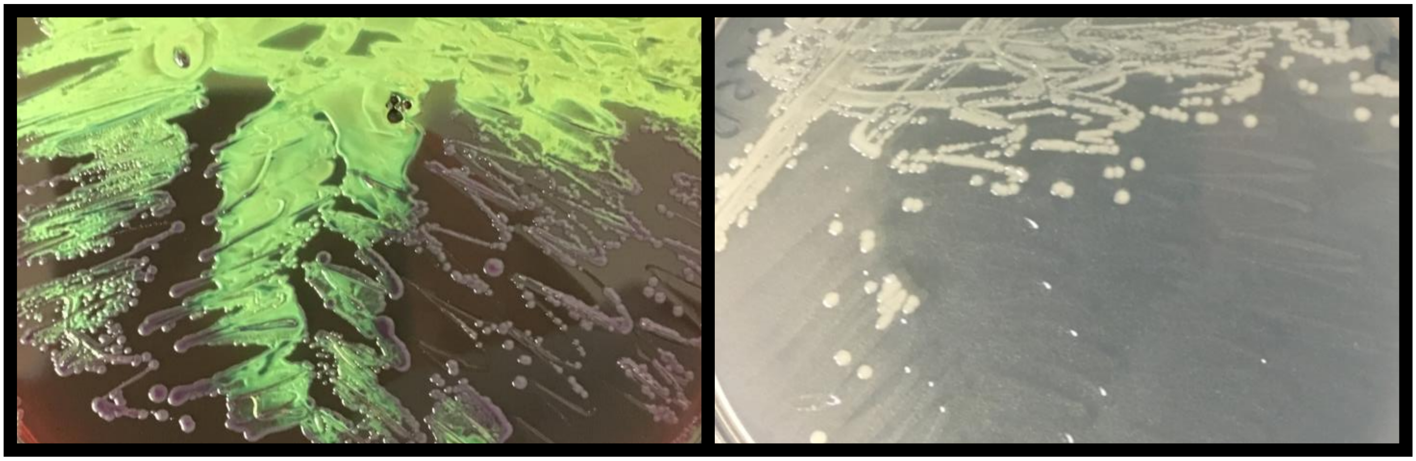

**Figure S3**. Colony Morphology of UCS1 *E. coli*. Clonal UCS1 colonies were streaked onto EMB agar (left) and LB agar (right) and incubated overnight at 37C.

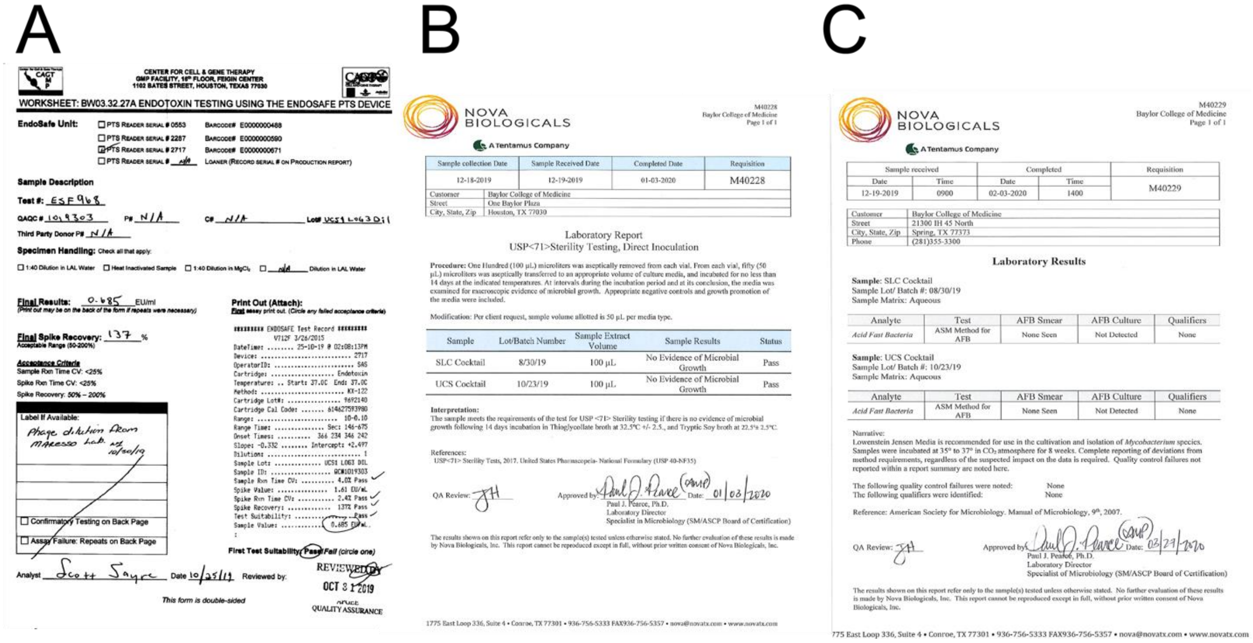
A

CENTER FOR CELL & GENE THERAPY
GMP FACILITY, 10th FLOOR, FEIGIN CENTER
1102 BATES STREET, HOUSTON, TEXAS 77030

WORKSHEET: BW03.32.27A ENDOTOXIN TESTING USING THE ENDOSAFE PTS DEVICE

EndoSafe Unit:
☐ PTS READER SERIAL # 0553 BARCODE# E0000000488
☐ PTS READER SERIAL # 2287 BARCODE# E0000000590
☑ PTS READER SERIAL # 2717 BARCODE# E0000000671
☐ PTS READER SERIAL # n/a LOANER (RECORD SERIAL # ON PRODUCTION REPORT)

Sample Description
Test #: ESF968
QAQC # 1019303 P# N/A C# N/A Lot# UCS1 Log3 Dil
Third Party Donor P# N/A

Specimen Handling: Check all that apply:
☐ 1:40 Dilution in LAL Water ☐ Heat Inactivated Sample ☐ 1:40 Dilution in $MgCl_2$ ☐ n/a Dilution in LAL Water

Final Results: 0.685 EU/ml
(Print out may be on the back of the form if repeats were necessary)

Final Spike Recovery: 137 %
Acceptable Range (50-200%)

Acceptance Criteria
Sample Rxn Time CV: <25%
Spike Rxn Time CV: <25%
Spike Recovery: 50% – 200%

Label If Available:
Phage dilution from Maresso Lab. at 10/30/19

☐ Confirmatory Testing on Back Page
☐ Assay Failure: Repeats on Back Page

Print Out (Attach):
First assay print out. (Circle any failed acceptance criteria)

```
######### ENDOSAFE Test Record #########
         V712F 3/26/2015
DateTime: ........ 25-10-19 @ 02:08:13PM
Device: ........................... 2717
OperatorID: ........................ SAS
Cartridge: .................. Endotoxin
Temperature: .. Start: 37.0C  End: 37.0C
Method: ......................... KX-122
Cartridge Lot#: ............... 9692140
Cartridge Cal Code: ...... 614627593980
Range: ........................ 10-0.10
Range Time: .............. Sec: 146-675
Onset Times: .........  366 234 346 242
Slope: -0.332 ........ Intercept: +2.497
Dilution: ............................ 1
Sample Lot: ............. UCS1 LOG3 DIL
Sample ID: ................. QC#1019303
Sample Rxn Time CV: .......... 4.0% Pass
Spike Value: ............... 1.61 EU/mL
Spike Rxn Time CV: ........... 2.4% Pass
Spike Recovery: .............. 137% Pass
Test Suitability: ................ Pass
Sample Value: ............. 0.685 EU/mL
:
```

First Test Suitability: Pass/Fail (circle one)

Analyst Scott Sayre Date 10/25/19 Reviewed by:
REVIEWED BY
OCT 3 1 2019
[illegible]
QUALITY ASSURANCE

This form is double-sided

B

NOVA BIOLOGICALS
A Tentamus Company

M40228
Baylor College of Medicine
Page 1 of 1

| Sample collection Date | Sample Received Date | Completed Date | Requisition |
|---|---|---|---|
| 12-18-2019 | 12-19-2019 | 01-03-2020 | M40228 |

| | |
|---|---|
| Customer | Baylor College of Medicine |
| Street | One Baylor Plaza |
| City, State, Zip | Houston, TX 77030 |

Laboratory Report
USP<71>Sterility Testing, Direct Inoculation

**Procedure:** One Hundred (100 µL) microliters was aseptically removed from each vial. From each vial, fifty (50 µL) microliters was aseptically transferred to an appropriate volume of culture media, and incubated for no less than 14 days at the indicated temperatures. At intervals during the incubation period and at its conclusion, the media was examined for macroscopic evidence of microbial growth. Appropriate negative controls and growth promotion of the media were included.

Modification: Per client request, sample volume allotted is 50 µL per media type.

| Sample | Lot/Batch Number | Sample Extract Volume | Sample Results | Status |
|---|---|---|---|---|
| SLC Cocktail | 8/30/19 | 100 µL | No Evidence of Microbial Growth | Pass |
| UCS Cocktail | 10/23/19 | 100 µL | No Evidence of Microbial Growth | Pass |

**Interpretation:**
The sample meets the requirements of the test for USP <71> Sterility testing if there is no evidence of microbial growth following 14 days incubation in Thioglycollate broth at 32.5°C +/- 2.5., and Tryptic Soy broth at 22.5°± 2.5°C.

References:
USP<71> Sterility Tests, 2017. United States Pharmacopeia- National Formulary (USP 40-NF35)

QA Review: JH
Approved by: (SAMP) Date: 01/03/2020
Paul J. Pearce, Ph.D.
Laboratory Director
Specialist in Microbiology (SM/ASCP Board of Certification)

The results shown on this report refer only to the sample(s) tested unless otherwise stated. No further evaluation of these results is made by Nova Biologicals, Inc. This report cannot be reproduced except in full, without prior written consent of Nova Biologicals, Inc.

1775 East Loop 336, Suite 4 • Conroe, TX 77301 • 936-756-5333 FAX936-756-5357 • nova@novatx.com • www.novatx.com

C

NOVA BIOLOGICALS
A Tentamus Company

M40229
Baylor College of Medicine
Page 1 of 1

| Sample received | | Completed | | Requisition |
|---|---|---|---|---|
| Date | Time | Date | Time | |
| 12-19-2019 | 0900 | 02-03-2020 | 1400 | M40229 |

| | |
|---|---|
| Customer | Baylor College of Medicine |
| Street | 21300 IH 45 North |
| City, State, Zip | Spring, TX 77373 |
| Phone | (281)355-3300 |

**Laboratory Results**

**Sample**: SLC Cocktail
Sample Lot/ Batch #: 08/30/19
Sample Matrix: Aqueous

| Analyte | Test | AFB Smear | AFB Culture | Qualifiers |
|---|---|---|---|---|
| *Acid Fast Bacteria* | ASM Method for AFB | None Seen | Not Detected | None |

**Sample**: UCS Cocktail
Sample Lot/ Batch #: 10/23/19
Sample Matrix: Aqueous

| Analyte | Test | AFB Smear | AFB Culture | Qualifiers |
|---|---|---|---|---|
| *Acid Fast Bacteria* | ASM Method for AFB | None Seen | Not Detected | None |

Narrative:
Lowenstein Jensen Media is recommended for use in the cultivation and isolation of *Mycobacterium* species. Samples were incubated at 35° to 37° in $CO_2$ atmosphere for 8 weeks. Complete reporting of deviations from method requirements, regardless of the suspected impact on the data is required. Quality control failures not reported within a report summary are noted here.

The following quality control failures were noted: None
The following qualifiers were identified: None

Reference: American Society for Microbiology. Manual of Microbiology, 9th, 2007.

QA Review: JH
Approved by: (SAMP) Date: 02/27/2020
Paul J. Pearce, Ph.D.
Laboratory Director
Specialist of Microbiology (SM/ASCP Board of Certification)

The results shown on this report refer only to the sample(s) tested unless otherwise stated. No further evaluation of these results is made by Nova Biologicals, Inc. This report cannot be reproduced except in full, without prior written consent of Nova Biologicals, Inc.

775 East Loop 336, Suite 4 • Conroe, TX 77301 • 936-756-5333 FAX936-756-5357 • nova@novatx.com • www.novatx.com

**Figure S4. Characterization of UCS1 cocktail by certified laboratories**. **A.** UCS1 phage cocktail was subjected to USP 85 endotoxin testing by limulus amoebocyte assay (Endosafe cartridges), **B.** USP 71 sterility testing by direct inoculation in rich media (Tryptic Soy Broth, Thioglycollate Broth), and **C.** Acid Fast Bacteria testing (Lowenstein Jensen Media)
